# Nitric oxide synthase and its function in animal reproduction: an update

**DOI:** 10.3389/fphys.2023.1288669

**Published:** 2023-11-07

**Authors:** Wei Zhang, Su juan Chen, Li ya Guo, Zijing Zhang, Jia bin Zhang, Xiao meng Wang, Xiang bo Meng, Min ying Zhang, Ke ke Zhang, Lin lin Chen, Yi wei Li, Yuliang Wen, Lei Wang, Jian he Hu, Yue yu Bai, Xiao jian Zhang

**Affiliations:** ^1^ College of Animal Science, Henan Institute of Science and Technology, Xinxiang, Henan, China; ^2^ Department of Life Science and Technology, Xinxiang Medical College, Xinxiang, Henan, China; ^3^ Institute of Animal Husbandry and Veterinary Science, Henan Academy of Agricultural Sciences, Zhengzhou, Henan, China; ^4^ College of Veterinary Medicine, Jilin Agriculture University, Changchun, China; ^5^ Animal Health Supervision in Henan Province, Zhengzhou, Henan, China

**Keywords:** nitric oxide synthase, structure, location, reproductive functions, regulation mechanism

## Abstract

Nitric oxide (NO), a free radical labile gas, is involved in the regulation of various biological functions and physiological processes during animal reproduction. Recently, increasing evidence suggests that the biological role and chemical fate of NO is dependent on dynamic regulation of its biosynthetic enzyme, three distinct nitric oxide synthase (NOS) according to their structure, location and function. The impact of NOS isoforms on reproductive functions need to be timely elucidated. Here, we focus on and the basic background and latest studies on the development, structure, importance inhibitor, location pattern, complex functions. Moreover, we summarize the exactly mechanisms which involved some cell signal pathways in the regulation of NOS with cellular and molecular level in the animal reproduction. Therefore, this growing research area provides the new insight into the important role of NOS male and female reproduction system. It also provides the treatment evidence on targeting NOS of reproductive regulation and diseases.

## Introduction

Over the last three decades, nitric oxide (NO), a small free-radical diatomic gas, has been identified as an extraordinarily important bioregulator that mediates a variety of biological functions in NO-synthesized cells and interactions with nearby cells and molecules (Hattori and Tabata, 2006; Iova et al., 2023). NO is synthesized by the oxidation of L-arginine-citrulline, which is mediated by nitric oxide synthase (NOS) and accompanied by NO production (Lind et al., 2017; Hosseini et al., 2022). The biological role and chemical fate of NO are affected by the subcellular localization of three distinct NOSs: neuronal NOS (nNOS), endothelial NOS (eNOS), and inducible NOS (iNOS) (Griffith and Stuehr, 1995; Alderton et al., 2001). In animal reproduction, there are several reports on the role of NOSs in terms of their abundance, location, isoforms, and activity and participates in complex physiological processes and pathological effects (Cinelli et al., 2020; Rostamzadeh et al., 2020; Solanki et al., 2022). However, there were lack of the exactly describe of NOS structure and summarize of the possible mechanisms which involved in the regulation of NOS in above publication. Therefore, we needed to pay more attention to the structure in different isoforms NOS which suited for the enzyme function and focus on more area abut male and female animal reproduction in this review.

### An overview of NOS from identified to now

Since the discovery of NO in 1980, NOS as the key synthase of NO has been explored and continue to be studied (Furchgott and Zawadzki, 1980; Palmer et al., 1987). In 1990, the NOSs were first identified and described as a 2′,5′-adenosine diphosphate affinity column eluted with nicotinamide adenine dinucleotide phosphate (NADPH) from rat cerebella. Thereafter, three major isoforms were molecularly cloned and purified over the next several years (Bredt and Snyder, 1990; Bredt et al., 1991; Schmidt and Murad, 1991).

The eNOS (NOS-3) enzyme, which comprises 26 exons, 25 introns, and 21–22 kbp at 7q35–7q36 of chromosome 7 from the vascular endothelium was identified in 1992 (Lamas et al., 1992; Marsden et al., 1992; Alderton et al., 2001). The iNOS (NOS-2) enzyme, which comprises 26 exons, 25 introns, and 37 kbp at 17cen–q11.2 of chromosome 17 was identified in human hepatocytes in 1993 (Geller et al., 1993). Notably, NOS-knockout mice were used in a functionality study in 1993 (Huang et al., 1993). The nNOS (NOS-1) enzyme, which comprises 29 exons, 28 introns and >200 kbp, at 12q24.2–12q24.3 of chromosome 12 was identified in 1994 (Hall et al., 1994). Furthermore, NOS recognition sites for NADPH, flavin-adenine dinucleotide (FAD), flavin mononucleotide, and calmodulin (CaM) indicate that the synthase is regulated by several factors (Bredt et al., 1991).

In animal reproduction, nitric oxide synthase activity in the male reproductive tract was found to be regulated by androgens (Chamness et al., 1995). The importance of the field of NO research was recognized in 1998 by the award of the Nobel Prize to Furchgott RF, Ignarro LJ and Murad F for their work that led to the discovery of NO as a biological mediator in mammalian cells. Considering the important roles of NO and NOS, inhibitors of the three NOS isoforms have received increasing attention in various fields of life sciences and have become a research hotspot.

In 2002, the first review on NOS inhibitors discussed iNOS inhibitors with classified, comprehensive information, and rational design (Vallance and Leiper., 2002). Moreover, exploration of novel domain architecture and functions revealed that tetrahydrobiopterin (BH4) serves as an electron donor in NOS oxygen activation and allows NOS to generate haem-oxy species that react with Arg or N-hydroxy-L-arginine (Stuehr et al., 2004; Stuehr and Haque., 2019). In addition, the role of heat shock protein 90 (hsp90) during NOS modulation and the exact mechanism of its regulation was discovered and applied in the development of several NOS isoform-specific drug prototypes that blocked dimerization of haem-containing NOS monomers in cells (Peng et al., 2012; Woodward et al., 2010).

**FIGURE 1:**
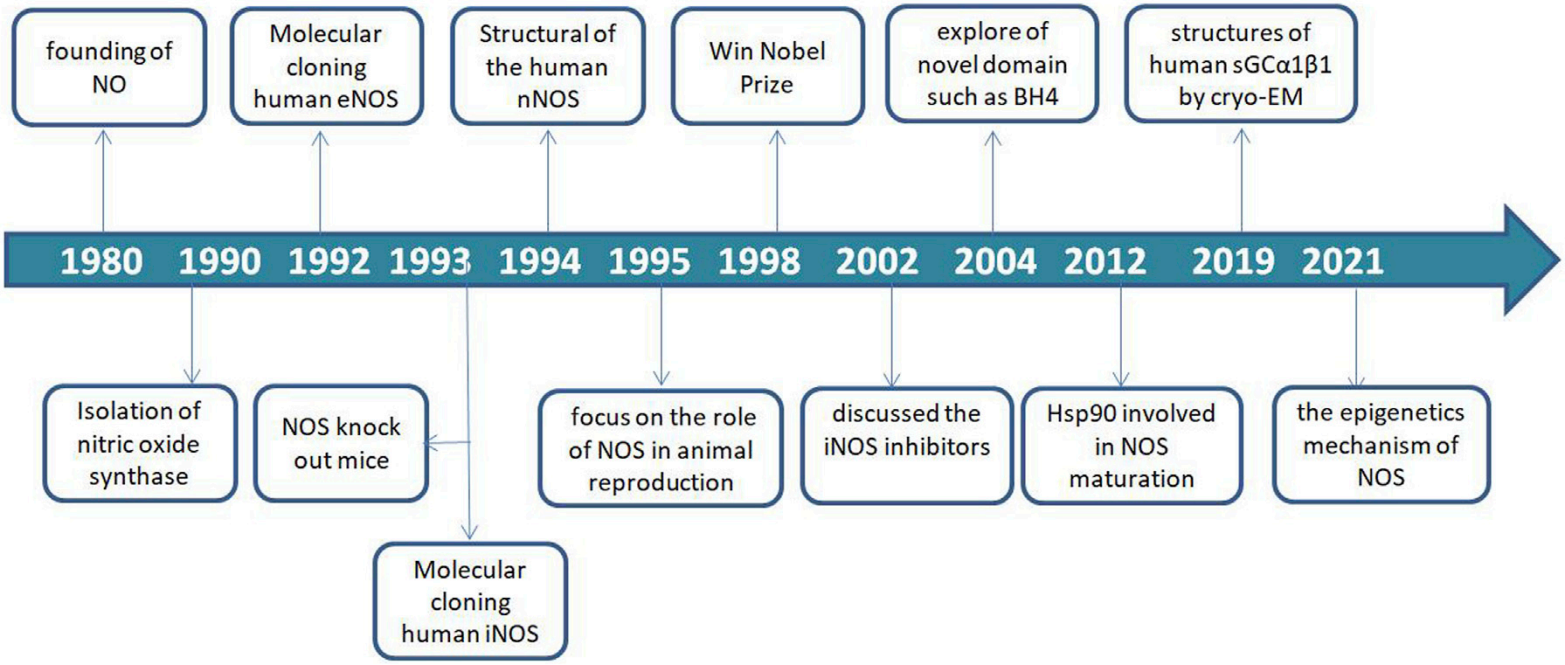
Timeline of nitric oxide synthase (NOS) development.

In recent years, nNOS and nNOS: CaM complexes have been investigated using cryogenic electron microscopy (cryo-EM). These investigations revealed the active-state architecture in which the nNOS reductase domains were identified to be flexibly linked in both CaM-free and CaM-bound states (Pospiech et al., 2019). Furthermore, the structure of human sGCα1β1, a key primary sensor of nitric oxide, revealed the transducer module bridges, the nitric oxide sensor module, and the catalytic module in different functional states using cryo-EM (Kang et al., 2019). Notably, the epigenetic mechanism plays a much greater role in nNOS than in other isoforms and induces the S-nitrosylation of histone deacetylase 2 relayed by the transnitrosylation of glyceraldehyde 3-phosphate dehydrogenase (Figure 1) (Yoon et al., 2021). Meanwhile, the overviews for NOS in different animal species was shown in Table 1.

**TABLE 1:** The overviews for NOS in different animal species.

Species	Breed	Isforms	Cell or tissue	Gene or protein feature	Enzyme charachteristics	Highlighting work	References
Mouse	No data	iNOS	macrophages	4.4 kb with 1,144 amino acids (aa) and the molecular mass 131KD	iNOS antigen only after exposure to IFN-y and LPS	Abnormal estrous cyclicity in disruption of eNOS and iNOS	Xie et al. (1992); Jablonka-Shariff et al. (1999)
	Balb/c mouse	nNOS	brain	4,388 bp and 1,429 aa, 97.9% and 93.6% identical to the rat and human	With recognition and consensus binding sites	generated mice that lack the nNOS gene but viable, fertile were normal	Ogura et al. (1993); Huang et al. (1993)
		eNOS	endothelial cells	4,140 bp and 1,202 aa	Contained with recognition and consensus binding sites	Endothelial nitric oxide deficiency results in abnormal placental metabolism	Lamas et al. (1992); George et al. (2022)
Rat	No data	iNOS	vascular smooth muscle cells	4 kb with 1,147 aa and the molecular mass 131KD	Contained NADPH, FMN, FAD binding regions	The different NOS were localization in the rat ovary during follicular development, ovulation and luteal formation	Nunokawa et al. (1993); Zackrisson et al. (1996)
	Wistar rats	nNOS	small intestine enteric nerve terminals	three different 5′-end splice variants of nNOS, 88% homology with exon 1 subtype from the mouse brain	Activity increased under NADPH (1 mM) and calmodulin (1 mM)	Invoved in neurotransmitters glutamate and nitric oxide during GnRH and LH release	Tatoyan and Giulivi (1998); Dhandapani and Brann (2000)
	Wistar rats	eNOS	Kidney endothelial cells	3,953 bp and 1,202 aa	No data	Involved in protective effects of L-carnitine on erectile function and reproductive function in diabetes rats	Mohaupt et al. (1994); Li et al. (2021)
	Wistar rats	mtNOS	liver mitochondria	120-130kD, highly unstable, dimeric under native conditions	activity increased 30%–40% associated with exogenous THB4,Ca^2+^, and calmodulin	No data	Tatoyan and Giulivi (1998)
Guinea pig	Hartley guinea-pig	iNOS	lung	3,447 bp, a protein of 1,149 residues with molecular mass of 131 kDa	concentration dependent manner with 100 nM calmodulin	iNOS to block implantation through action on the endometrium	Shirato et al. (1998), Shi et al. (2003)
Bovine	No data	iNOS	Alveolar macrophages	3,471 bp transcript, translated a protein of 1,156 aa	No data	Addition of NO can be avoided the free radical-induced damage	Widdison et al. (2007), Upadhyay et al. (2022)
	No data	nNOS	endothelial cells	4 kb with 1,232 aa	Activity increased under NADPH and calmodulin	Involved in the effect of endothelins on bovine oviductal and smooth muscle motility	Lamas et al. (1992); Kobayashi et al. (2016)
	No data	eNOS	Bovine aortic endothelial cells	3,650 bp, 58% and 51% identical to the rat and mouse	Calcium stimulated production of nitrite was enhanced under eNOS	Regulating final follicle maturation, ovulation and early luteal angiogenesis	Nishida et al. (1992), Berisha et al. (2020)
Pig	Yorkshire pigs	iNOS	Alveolar macrophages	3,948 bp, 1,064 aa,90.2% amino acid sequence identity with human and murine iNOS	No data	melatonin and silymarin can decrease ROS and NO production in frozen-thawed sperm via iNOS	Pampusch et al. (1998); Lee and Lee (2023)
	No data	eNOS	Pulmonary artery endothelial cells	representing a protein of 1,205 aa with a molecular mass of 134 kDa	Level of nitrite not increased after infection with PRSS virus	produced pigs carrying an eNOS gene driven by Tie-2 promoter and tagged with V5 His tag	Zhang et al. (1997); Hao et al. (2006)
	No data	nNOS	Kidneys of preweanling piglets	1,468 aa and 160kD, 76% homology human nNOS	No data	Involved in the effect of E_2_ levels on the cholinergic innervation pattern of ovaries during pathological states	Solhaug et al. (2000); Jana et al. (2018)
Sheep	No data	iNOS	white blood cells	4,192 bp and 1,154 aa, 88.8% homologous to the human protein	binding sites for calmodulin, FAD, FMN, NADPH-ribose, and NADPH-adenine	Involved in the arginine supplementation may accelerate ovulatory processes and the estrous rate	Mershon John et al. (2002); Guo et al. (2017)
	No data	nNOS	cerebella and cortex	150 kDa which correlates well with the other purified nNOS	Km (L-arginine) = 2.8 μM, Ec50 (CaCl_2_) = 280 nM	nNOS Involved in the release pattern of GnRH by the hypothalamus includes both pulses and surges	Crack et al. (1998); Virginia et al. (2021)
	No data	eNOS	Coronary artery tissues	4,097 bp and 1,205 aa, strong homology with the human eNOS	contains the cofactor binding sites at the amino terminal end	accelerate ovulatory processes and the estrous rate after arginine supplementation	Mershon John et al. (2002); Guo et al. (2017)
Poultry	Pekin ducks	iNOS	leukocytes from spleens	3,447 bp, encoded a protein of 1,148 aa with molecular weight of 130 kDa	iNOS levels can be elevated addition of LPS or IFN-γ in splenocyte cell culture	As an inflammatory factor during cage stress	Simon et al. (2011); Zhang et al. (2019)
	chickens	iNOS	chicken macrophage cell line, HD11	4.5kb and 1,136 amino acid, 66.6%, 70.4%, with mouse and human	pyrrolidine dithiocarbamate blocked substantially the accumulation of iNOS mRNA in chicken macrophage cells	Se effectively alleviated HgCl_2_-induced testes injury by p38 MAPK/ATF2/iNOS signaling pathway in chicken	Lin et al. (1996); Chen et al. (2022)

### The structure of NOSS

**FIGURE 2:**
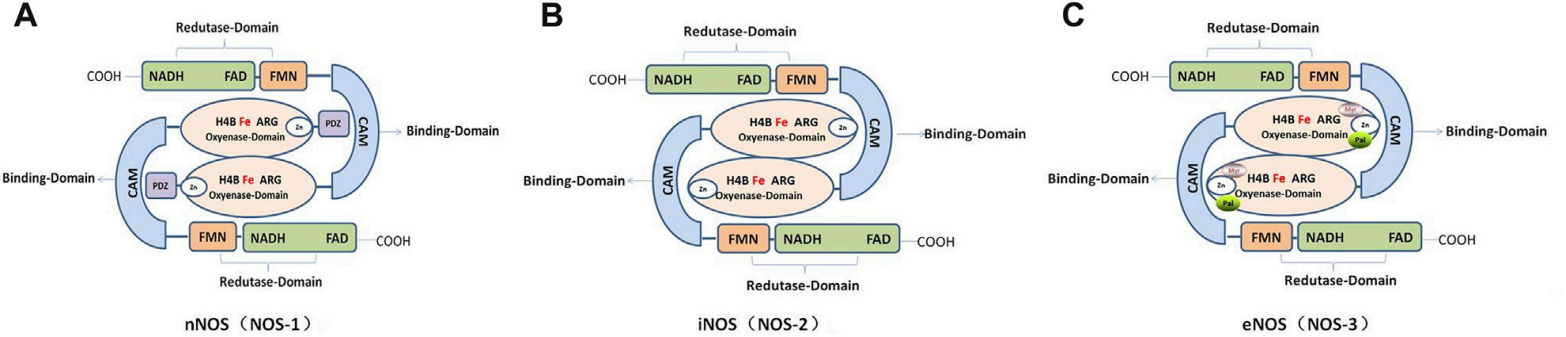
Schematic and functional domain structure of human nitric oxide synthase (NOS). **(A)**. Human neuronal NOS (nNOS; NOS-1). **(B)**. Human inducible NOS (Inos; NOS-2). **(C)**. Human endothelial NOS (eNOS; NOS-3).

The NOS monomer consists of a heme domain, BH4 bound to its N-terminus, and a reductase domain containing FAD and flavin-adenine mononucleotide (FMN) (Lind et al., 2017; Hosseini et al., 2022). The NOSs share homology in regions involved in cofactor binding, such as NADPH, FAD, FMN, CaM, and adenine binding sites. In addition, they have similar enzymatic mechanisms that involve electron transfer for the oxidation of the terminal guanidino nitrogen of L-arginine (Figure 2).

In mammals, NOSs can be classified into three different subtypes according to their position: neurons (nNOS, also known as NOS-1); immune system (iNOS or NOS-2); and endothelial cells (eNOS or NOS-3), which are involved in nerve transmission, smooth tissue relaxation, and immune responses, respectively. In addition, variants of other canonical isoforms include mitochondrial NOS (mtNOS) in the mitochondria (Carnicer et al., 2021; Carnicer et al., 2013; Li et al., 2014), penile NOS (PnNOS), testis-specific NOS (TnNOS), and invertebrate *Drosophila* NOS (dNOS) (Hosseini et al., 2022).

However, a highly conserved primary sequence (>90%) of the same NOS isoform from different mammalian species has been reported. For example, the structure of human eNOS highly resembles that of bovine eNOS in the same space group with one dimer per asymmetric unit with visible residues Lys67–Trp480 in the human eNOS structure, but Lys69–Trp482 in the bovine eNOS (Li et al., 2014).

### Neuronal nitric oxide synthase (NNOS)

The nNOS enzyme exists in two forms, i.e., soluble and particulate. The enzyme was originally identified in neurons, where it is concentrated at neuronal synapses. Human nNOS mainly contains the following: a PDZ domain at the NH2 terminus; an oxygenase domain containing heme and BH4 interacting sites; a reductase domain containing interacting sites for FMN, FAD, and NADPH; and the FMN domain connects to the oxygenase domain via the CaM domain. Furthermore, nNOS contains an auto-inhibition segment that interrupts the FMN domain (Carnicer et al., 2021; Solanki et al., 2022). In addition, there are five different isoforms of nNOS proteins that are products of alternatively spliced NOS1 mRNAs: nNOS-a, m, β, γ, and nNOS2 (Carnicer et al., 2013). Phosphorylation of nNOS at Ser847 by CaMKII and 90-kDa ribosomal S6 kinase 1 attenuates the NO synthesis activity of nNOS *in vitro* and in cells (Araki et al., 2020).

### Endothelial nitric oxide synthase (ENOS)

Compared with the nNOS and iNOS, eNOS proteins contain an autoinhibition segment that interrupts the FMN domain, and there is an oxygenase domain at the myristoylation, palmitoylation, and zinc-ligating positions (Solanki et al., 2022). In different species, the crystalline structure of human eNOS highly resembles that of bovine eNOS, with one dimer per asymmetric unit in the same space group, especially in the active sites of Asn366 and Val104 residues in human eNOS and Asn368 and Val106 in bovine eNOS. However, quite a few clusters of positive density in human eNOS appeared next to acidic residues on the protein surface that provided additional crystal contacts (Li et al., 2014; Li et al., 2014). CaM and hsp90 bind to eNOS and regulate its activity. CaM is induced by an increase in intracellular Ca^2+^ when phosphorylated at serine, threonine, and tyrosine residues, which stimulates the flux of electrons within the reductase domain and increase Ca^2+^ sensitivity of eNOS (Fleming and Busse, 2003). However, hsp90 is an allosteric modulator that activates the enzyme and promotes eNOS recoupling (Carnicer et al., 2013; Li et al., 2014; Man et al., 2022).

### Inducible nitric oxide synthase (INOS)

Notably, iNOS is the simplest mammalian NOS, consisting solely of reductase, oxygenase, and CaM-binding domains that lack the auto-inhibitory loop present in eNOS as well as the PDZ domain of nNOS (Cinelli et al., 2020). Due to its short half-life, iNOS is independent of calcium signaling and produces high concentrations in short pulses (Gage and Thippeswamy, 2021). In particular, the tight binding of CaM to iNOS allows it to be activated at low physiological concentrations of calcium (40 nM in iNOS vs. 400 nM in nNOS and eNOS). However, it is effectively locked in an active position where calcium regulation is no longer relevant (Cinelli et al., 2020; Venema et al., 1996). Furthermore, low doses of the essential cofactor BH4 may affect iNOS activity and dimerization and NO itself may negatively regulate iNOS activity (Assreuy et al., 1993). NOS expression can be induced by cytokines, environmental agents, and other diseases in almost all cell types, especially in inflammatory or abnormal conditions such as testicular injury or luteolysis (Cinelli et al., 2020; Fleming, 2010; Rostamzadeh et al., 2020). With regards to its expression and localization, iNOS is not constitutively expressed in cells, but its expression can be induced by infection, bacterial lipopolysaccharide (LPS), cytokines, and other agonists (Solanki et al., 2022).

### Other identified NOS isoforms

Several studies have reported the variants of other canonical isoforms in various tissues and organs. The mtNOS enzyme was first found in the matrix of the mitochondrial inner membrane using protein mass fingerprinting and anti-iNOS and anti-nNOS antibodies (Ghafourifar and Richter, 1997). Furthermore, the main biological function of mtNOS is to catalyze the production of NO and inhibit the uptake of Ca^2+^ and increase mitochondrial Ca^2+^ by negative feedback, which maintains the stability of intracellular Ca^2+^. However, further studies are required to clarify whether mtNOS is an independent gene entity or a product of three conventional NOS-encoding genes (Lacza et al., 2009; Shvedova et al., 2018).

The pnNOS enzyme is an nNOS variant expressed in the penis and prostate and exists as alpha and beta spliceoforms that lack an N-terminal PDZ domain. The alpha splice variant is active in NO formation at nerve terminals, whereas the functional role of the beta variant *in vivo* is unclear and may not be substantial (Musicki et al., 2009; Musicki et al., 2011).

Testis-specific NOS (TnNOS) is a variant of the nNOS protein, the mRNA expression of which is restricted in male gonadal tissues, especially in Leydig cells. TnNOS is a 125-kd protein and possesses NOS enzymatic activity comparable to that of the full-length nNOS (160 kd). Moreover, this protein variant lacks the PDZ protein interaction domain implicated in membrane localization. TnNOS may have a unique biological role in the testes or play an important role in the regulation of testosterone release and represents an intriguing model (Hosseini et al., 2022; Wang et al., 2002).

### NOS inhibitors

Given their importance, there is a need to understand the structural and pharmacophoric requirements for the development of potent and selective NOS inhibitors based on NOS structure and function for potential clinical use. NOS inhibitors are broadly classified into two categories depending on their source or origin, that is, natural and synthetic (Table 2). Synthetic and highly selective NOS inhibitors are classified into arginine and non-arginine analogs which classified base on pharmacophore, functional groups, or heterocyclic ring present (Król and Kepinska et al., 2020; Minhas et al., 2020; Sakamuri et al., 2020). NOS inhibitors were first designed in the 1980s and the 1990s and are based on L-arginine, an enzyme substrate. This approach yielded strong compounds, but unfortunately, with a poor level of selectivity among the isoforms. By the end of the 1990s, the first crystal structures of iNOS and eNOS showed a high degree of similarity, particularly with respect to their active sites. One of the most critical moments in the history of NOS inhibitors was the description of highly selective iNOS inhibitors (Minhas et al., 2020; Oliveira et al., 2013; Pradhan et al., 2018). However, many NOS inhibitors have been produced:

**TABLE 2:** The different inhibitors of nNOS and iNOS.

NOS isforms	Inhibitor name	Chemical constitution	IC 50 or Ki	Application	References
nNOS	disubstituted indoline derivatives	disubstituted indoline derivatives	IC 50 = 0.37 µM	In neuronal cells and many kinds of neurodegenerative disorders	Annedi et al. (2012)
	3,4-dihydroquinolin-2(1H)-one and 1,2,3,4-tetra-hydroquinoline	derivatives of quinoline	IC 50 = 0.089 µM	Reverse thermal hyperalgesia and reduce tactile hyperesthesia in rats with a dose of 30 mg/kg	Ramnauth et al. (2011); Yang et al. (2015)
	2-amino-4-methylpyridine groups with a chiral pyrrolidine linker	Pyrrolidine derivatives	Ki = 9.7 nM	against three different isoforms of NOS, including rat nNOS, bovine eNOS, and murine macrophage iNOS	Jing et al. (2014)
	a-amino	Aminopyridine derivatives	Ki = 24 nM	with 273- and 2822-fold selectivity against iNOS and eNOS	Kang et al. (2014)
	benzo [d]thiazol-2-yl-methyl-4-(substituted)-piperazine-1-carbothioamide	benzothiazole-piperazine carbothioamide	Inhibition activity (nNOS = 66.73 ± 1.51)	6-OHDA-induced unilaterally lesioned rats showed the improvement in motor and non-motor functions with significant nNOS binding affinity	Agrawal et al. (2022)
	ZINC000013485422	4,6-bis (3- methylbut-2-en-1-yl) −8,17-dioxatetracyclo [8.7.0.0 2, 7 .011, 16 ]heptadeca-2,4,6,11,13,15-hexaene-5,14-diol	Ki = 114.06 nM	No data	Boumezber and Yelekci (2023)
iNOS	four farnesyl phenols (grifolinones)	Polyphenolic constituents	IC 50 = 23.4, 22.9, 29.0, and 23.3µM, respectively	Inhibit NO production in RAW-264.7 cells	Quang et al. (2006)
	narchinol C	sesquiterpenoids	IC 50 = 21.6 µM		Guo et al. (2019)
	L-NAME	arginine analogs	IC 50 = 20 μM, Ki = 3.9 µM	Inhibit NO production in many cell	Cheshire et al. (2011); Cinelli et al. (2020)
	acetamidine derivatives	acetamidine derivatives	IC 50 = 53 µM	Inhibit NO production in glioma cells	Fantacuzzi et al. (2016)
	Nitroguanidinoalkylamide of five-membered heterocyclic compounds	five-membered heterocyclic compounds	IC 50 = 2.36 µM	Inhibitory effects of test samples on the NO production in LPS-activated mouse macrophages	Liu et al. (2008)
	Carbothioamide analog of hexahy dropyridazine 1-carboximidamides	Six-membered heterocyclic compounds	IC 50 = 0.6 mM	Inhibit NO production in RIN-5H cells	Minhas et al. (2020); Morgenstern et al. (2004)
	derivatives of glycyrrhetinic acid	Steroidal compounds	IC 50 = 50 µM	evaluated the anti-inflammatory activity in LPS-induced RAW-264.7 macrophage cells	Chiaradia et al. (2008); You et al. (2013)

Among the inhibitors of nNOS, one of the disubstituted indoline derivatives from Neur Axon has a bulky cyclic amino-substituent-4-(methylamino) cyclohexyl group at the 1-position of the indoline and presents the best selectivity (IC_50_ = 0.37) (Annedi et al., 2012). The 3,4-dihydroquinolin-2(1H)-one and 1,2,3,4-tetra-hydroquinolineinhibitors are derivatives of quinoline and contain a 6-substituted thiophene amidine group with excellent potency and selectivity for nNOS (IC_50_ = 0.089) (Ramnauth et al., 2011; Yang et al., 2015). Furthermore, pyrrolidine derivatives containing one or two 2-amino-4-methylpyridine groups with a chiral pyrrolidine linker exhibited the best activity and potency of 9.7 nM (Jing et al., 2014). Several a-amino functionalized aminopyridine derivatives were designed to target to BH4 against nNOS, exhibiting a K_i_ of 24 nM for nNOS, with 273 and 2822-fold selectivity against iNOS and eNOS, respectively (Kang et al., 2014).

In recent years, accompanying structure-based virtual screening, molecular docking, and molecular dynamics simulation drug design techniques, benzo [d]thiazol-2-yl-methyl-4-(substituted)-piperazine-1-carbothioamide was synthesized as a novel nNOS inhibitor, showing the highest selectivity for nNOS (nNOS = 66.73 ± 1.51; eNOS = 28.70 ± 1.39; iNOS = 13.26 ± 1.01) in HEK 293 cells expressing NOS isoforms compared with 7-nitroindazole (7-NI), a widely accepted nNOS inhibitor in the animal models (Agrawal et al., 2022). ZINC000013485422 showed good stability and selectivity for nNOS through dual van der Waals interactions, multiple alkyl interactions, and one pi-cation interaction formed with nNOS, which kept the molecule firmly attached to the target (Boumezber and Yelekçi., 2023).

In inhibitors of iNOS, four farnesyl phenols (grifolinones A and B, grifolin, and neogrifolin) extracted from an inedible mushroom, *Albatrellus caeruleoporus*, exhibited inhibitory activity (IC_50_ = 23.4, 22.9, 29.0, and 23.3 µM, respectively) significantly higher than that of L-NMA (IC_50_ 88.4 µM) (Quang et al., 2006). Narchinol C, a sesquiterpenoid, inhibited NO production (IC_50_ = 21.6 µM) in RAW-264.7 cells, comparable to aminoguanidine (IC_50_ = 17.5 µM) (Guo et al., 2019). There are many synthetic molecules for inhibiting iNOS directly (Cheshire et al., 2011; Cinelli et al., 2020). Of all synthesized acetamidine derivatives, compounds with an indole ring substituted with an acetamidino group through a methylene linker maximally inhibited iNOS (IC_50_ = 53 nM) with good selectivity in a recombinant enzyme assay of murine iNOS (Fantacuzzi et al., 2016). Furthermore, nitroguanidinoalkylamide coupled to variedly substituted phenyl ring of five-membered heterocyclic compounds through pyrrolidine, yielded an unsubstituted phenyl analog 122 (R = H) and was identified as the most active iNOS inhibitor (IC_50_ = 2.36 µM) in an isolated enzyme assay (Liu et al., 2008).

In addition, 1-carbothioamide analog of hexahy dropyridazine-1-carboximidamides, a six-membered heterocyclic compound, completely inhibited the production of NO (IC_50_ = 0.6 mM) in RIN-5H cells (Minhas et al., 2020; Morgenstern et al., 2004). Several oxadiazole derivatives, such as 3-pyridyl, are potent iNOS inhibitors (IC_50_ = 0.05 µM) that bind iNOS through its pyridyl nitrogen projecting toward Gly391, through hydrogen bonding with Gln190, Gly372, Gly391, and Gly406 as well as four cation-π interactions (Sun et al., 2011). Furthermore, steroidal compounds (derivatives of glycyrrhetinic acid) and chalcone derivatives (2, 4, 6-trimethoxyacetophenone) have been evaluate for their anti-inflammatory activity in LPS-induced RAW-264.7 macrophage cells with inhibition at 50 μM and 27.60 μM respectively (Chiaradia et al., 2008; You et al., 2013). In general, when people develop potent inhibitors of nNOS or iNOS, the high selectivity of these inhibitors for nNOS and iNOS over eNOS is critically important. Furthermore, because eNOS is found predominantly in the vascular endothelium and is fundamental for healthy cardiovascular function, the inhibition of eNOS is very likely to produce unwanted side effects. Thus, no patents have been reported for eNOS inhibitors (Pradhan et al., 2018; Yang et al., 2015).

## Location of NOS in animal reproductive system

### Location of NOS in male animal reproductive system

Considering the abundant role of NOS, different isoforms are found in the epithelium, muscle, endothelium, and neurons of the male animal reproductive system. All three NOS isoforms are found in the testes and display distinctive yet overlapping cellular distribution patterns. Specifically, nNOS, iNOS, and eNOS are found in both Sertoli and germ cells in the seminiferous epithelium, in myoid cells, in endothelial cells, in myofibroblasts, and in spermatozoa and Leydig cells (Fujisawa et al., 2000; Lue et al., 2003; Zini et al., 1999). Remarkably, a testis-specific, truncated form of nNOS (TnNOS) has recently been shown to localize exclusively in Leydig cells but not in Sertoli and germ cells (Davidoff et al., 1997; Lissbrant et al., 1997; Tatsumi et al., 1997), indicating a potential role in steroidogenesis. Furthermore, it is not apparent whether these NOSs are stage-specific proteins in the seminiferous epithelium throughout the epithelial cycle (Lee and Cheng, 2004).

### Location of NOS in female animal reproductive system

In the female animal reproductive system, the expression and activity of NOS greatly depends on the cell type, ovarian vascular system, resident or infiltrating macrophages, and animal species. Moreover, it varies throughout different ovarian processes (Budani and Tiboni, 2021; Iova et al., 2023; Rosselli et al., 1998).

In the ovary, eNOS was expressed in granulosaluteal cells, rat mural granulosa cells (Jablonka-Shariff and Olson., 1997), blood vessels (Van Voorhis et al., 1995), rat stroma, thecal and luteal cells (Zackrisson et al., 1996), cattle granulosa cells (Pires et al., 2009), the theca, granulosa and cumulus cells of buffalo, ovarian preantral follicles (PFs), antral follicles (AFs) and ovulatory follicles (OFs) (Dubey et al., 2012), the endothelium, chorioallantoic membrane, luminal and glandular epithelium of ovine placental and uterine tissues (Zheng et al., 2000), the equine endometrium (Roberto da Costa et al., 2007), and pig granulosa cells (Kim et al., 2005; Ponderato et al., 2000). In addition, iNOS is expressed in rat granulosa cells from primary, secondary, and small antral follicles (Van Voorhis et al., 1995); rat stroma, thecal, and luteal cells; immature and *in vitro* matured oocytes of cattle (Pires et al., 2009; Zackrisson et al., 1996; Zamberlam et al., 2011); and granulosa and theca cells in buffalo (Dubey et al., 2012).

In addition, in the placenta, iNOS is expressed in macrophages, the immune system, and other cells, but is also found in endothelial cells and trophoblast cells; however, this isoform has not been detected in porcine granulosa cells (Jablonka-Shariff and Olson., 1997; Ponderato et al., 2000). In early embryo development, NO production has also been demonstrated in bovine embryonic stem cells of the blastocyst cell mass and in the placenta, cytotrophoblasts, and syncytium in the placental vascular wall in the second trimester of pregnancy (Basini et al., 1998; Basini and Grasselli, 2000).

Although nNOS has the same location pattern as iNOS and eNOS, nNOS is expressed in porcine granulosa cells in the theca, granulosa, and cumulus cells of PFs, AFs, and OFs, and appears to be involved in oocyte maturation or activation with weak immunohistochemistry (Dubey et al., 2012; Kim et al., 2005; Nakamura et al., 2002; Petr et al., 2006). However, on postnatal days 1, 5, 7, 10, and 19 in rats, all three isoforms of NOS were mainly localized to the oocytes and were expressed as a gradual increase in granulosa cells and theca cells within the growing follicle. The ovarian total NOS activity and NO levels increased on postnatal days 7 and 10 compared with other days (Zhang et al., 2011). In fetal and neonatal pigs, all three isoforms of NOS are mainly localized in the oocyte and show a gradual increase in granulosa and theca cells with growing follicles (Ding et al., 2012).

### The role of NOS in animal reproduction

**FIGURE 3:**
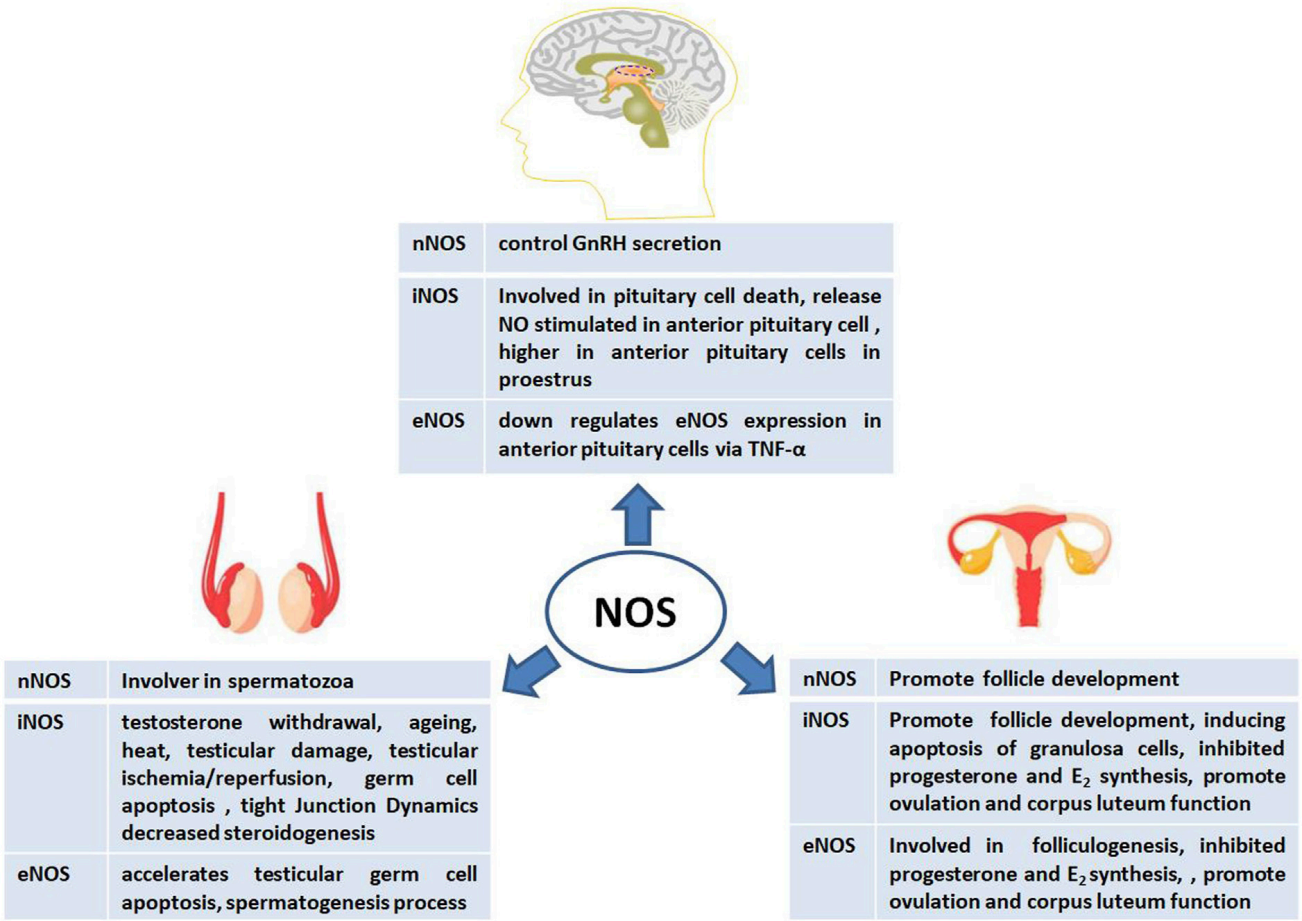
Physiological and pathological effects of nitric oxide synthase (NOS) on male and female animal reproductive functions.

To date, several reports on the role of NOS in animal reproduction have been published owing to its abundant location, isoforms, and activity. During animal reproduction, NOS exerts regulatory effects on the central nervous system, including the pituitary gland. In males, NOS is involved in spermatogenesis, the testis, the blood-testis barrier (BTB), and steroidogenesis. In females, NOS also participates in complex physiological processes such as follicle development, steroidogenesis, and ovulation (Figure 3). Interestingly, the exact physiological and pathological effects of NOS on male and female reproductive functions are based on its isoforms.

## The role of NOS in male animal reproduction

### The role of NOS in spermatogenesis and the testis

In the testis, the NO–NOS system is upregulated under different stimuli, such as testosterone withdrawal, aging, and heat, which generate testicular reactive oxygen species (Turner and Lysiak, 2008). For example, in testicular torsion model, the expression of iNOS and NO were increased but iNOS inhibitors can protect against testicular injury (Shiraishi et al., 2001). NOS is a vital factor in testicular damage induced by different conditions. For example, increased eNOS and iNOS expression and decreased nNOS expression were detected in a testicular damage-induced diabetic group; however, leptin partially prevented testicular damage by ameliorating histopathological changes by suppressing iNOS expression (Kapucu and Akgun-Dar, 2021). Moreover, Selenium (Se) effectively alleviates HgCl_2_-induced testicular injury by improving the antioxidant capacity to reduce inflammation mediated by the p38 mitogen-activated protein kinase (MAPK)/activating transcription factor 2 (ATF2)/iNOS signaling pathway in chickens by decreasing iNOS protein expression (Chen et al., 2022). The iNOS protein also increased testicular ischemia/reperfusion (I/R) in injured rats and induced spermatogenetic activity, seminiferous tubular diameter, and Leydig cell mass (Sangodele et al., 2021). Therefore, iNOS is involved in testicular damage via decreased tissue antioxidative defense capacity and the regulation of testicular hemodynamics (El-Shalofy et al., 2023).

NOSs have also been shown to regulate germ cell development in the testis (Zini et al., 1996; O’Bryan et al., 1998; Zini et al., 1998; Taneli et al., 2005; Dutta et al., 2022). Remarkably, the first implication of NO in sperm motility stems from localization studies, which demonstrated the presence of all three types of NOS in spermatozoa (Herrero et al., 1996; Revelli et al., 1999; Zini et al., 1998). The iNOS protein may play a role in regulation of germ cell apoptosis in testis by treatment of other factors such as the environment (heat), reproductive hormones (testosterone, follicle-stimulating hormone) or compounds (astragalin). To prevent spermatogenic dysfunction it downregulates the protein expressions of tumor necrosis factor (TNF)-α and iNOS but not eNOS in testes which increases antioxidant enzyme activities and inhibits inflammation (Guo et al., 2009; Han et al., 2019; Olfati and Moghaddam, 2018).

The eNOS enzyme plays an important role in spermatogenesis. When eNOS was overexpressed in transgenic mice, the numbers of spermatocytes and spermatids in eNOS-Tg cryptorchid testes significantly decreased compared with those in wild-type cryptorchid testes from day 3, This suggested accelerated testicular germ cell apoptosis induced by experimental cryptorchidism (Ishikawa et al., 2005). In addition, treatment of spermatozoa with NOS inhibitors leads to reduced motility, and the T allele, which encodes aspartic acid, of the eNOS (Glu298Asp) 248 single nucleotide polymorphism (SNP) may be associated with low sperm motility via activation of the cyclic guanosine monophosphate (GMP)/protein kinase G signaling pathway (Buldreghini et al., 2010; Lewis et al., 1996; Miraglia et al., 2011). Abdelzaher’s research shows that an increase in eNOS activity may help nicorandil to protect against testicular toxicity, such as reduction in the number of germinal epithelium, sloughed germinal cells with pyknotic nuclei, and arrest of spermatogenesis induced by methotrexate (MTX). MTX can also reduce the immune expression of eNOS in testicular tissue (Abdelzaher et al., 2020). Therefore, eNOS is a cytoplasmic protein that supports cells and interstitial cells, spermatogenesis, the vas deferens, and the epididymal epithelium, suggesting a crucial role of NO/NOS in the normal functioning of spermatozoa.

In addition, eNOS and iNOS reactions were considerably higher in the spermatozoa-present group than in the spermatozoa-absent group, but the nNOS reaction was only prominent in the Leydig cells in both groups. These results suggested that eNOS, iNOS, and mast cells play important roles in spermatogenesis (Hürda et al., 2021). However, NOS systems are upregulated in models of testicular damage and in human testes with maturation arrest and may contribute to the impairment of spermatogenesis by preventing adequate functioning of the spermatogonia population via impaired spermatogonia cell cycle, thereby inducing GC-1 arrest in the S phase (Ferreiro, 2019). Furthermore, supplementation with arginine significantly increased serum NO levels in 150-day-old boars, along with a significant increase in total nitric oxide synthase activity, demonstrating that additional arginine supplementation in the diet can increase serum NO levels (Wei et al., 2022). NOS systems are primarily based on iNOS and eNOS, which are involved in spermatogenesis and testicular development. However, the exact role of nNOS requires further investigation.

### The role of NOS in tight junction dynamics and adherens junctions

NOS is involved in the connections between Sertoli and germ cells in the BTB (Rostamzadeh et al., 2020). Sertoli cell tight junctions (TJs) are located near the basal lamina of the testes and are closest to the basement membrane. NO/NOS signaling pathways that are known to regulate Sertoli cell TJ dynamics are as follows: First, NO stimulates soluble guanylyl cyclase (sGC) to synthesize cyclic (cGMP), leading to TJ disruption and activation of protein kinase G (PKG). In turn, this can affect TJ dynamics via its effects on occludin, reducing the level of occludin at the site of Sertoli cell TJ, thereby opening up the TJ barrier (Lee and Cheng, 2003; Lee and Cheng, 2004; Lee and Cheng, 2008). Furthermore, it is apparent that the effects of NOS on the permeability barrier are based on testicular conditions (Kubes, 1995; Michel and Curry, 1999). For example, NOS, especially iNOS, is upregulated in the testes of rats with autoimmune orchitis, leading to testicular impairment (Ni et al., 2019; Jarazo Dietrich et al., 2012).

In the testis, adhesion between Sertoli cells and spermatids is conferred by cell-cell actin-based adherens junctions (AJs) (Cheng and Mruk., 1985; Russell and Peterson., 1985). AJs are found at the Sertoli-Sertoli and Sertoli-germ cell interfaces in the epithelium from the basal to the adluminal compartment, depending on ectoplasmic specialization in the testis, in an *in vivo* model in which adult rats were treated with adjudin, a molecule that induces adherens junction disruption.

Disruption of AJ is also associated with transient iNOS induction. Immunohistochemistry showed that iNOS accumulated intensely in the Sertoli and germ cells in the epithelium during adjudin-induced germ cell loss, with concomitant accumulation of intracellular cGMP and induction of PRKG, but not cyclic adenosine monophosphate (cAMP) or protein kinase A (PKA). Therefore, NOS/NO regulates Sertoli germ cell AJ dynamics via the cGMP/PRKG pathway (Lee et al., 2005). Lee et al. further found that eNOS and iNOS may depend on the downstream sGC/cGMP/protein kinase G signaling pathway to regulate the structural components of tight junctions and adhesive junctions of the testis, leading to spermatogenic epithelium (Wang et al., 2020).

### The role of NOS in male steroidogenesis

Male steroidogenesis is based on three levels of the hypothalamic-pituitary-gonadal axis and steroidogenic cells within the adrenal cortex and gonads via the steroidogenic pathway. Abundant evidence suggests that NOS plays an important role in the control of reproduction because of its ability to regulate gonadotropin-releasing hormone (GnRH) secretion from the hypothalamus and pituitary gonadotropes (Mondillo ett al., 2009). Briefly, NO produced by NOS can activate cGMP-dependent protein kinase 1(PRKG1) and the phosphorylation of astrocytes to activate soluble cGMP, thereby promoting steroid production in Leydig cells. In 2002, Drewett et al. showed that NO can inhibit cytochrome P450scc (CYP11A1) at higher doses, thus inhibiting steroid production in Leydig cells (Wei et al., 2002). Testicular cells are well-equipped with a NO-cGMP pathway, which may significantly participate in the regulation of testicular functions, such as spermatogenesis and steroidogenesis, by reversibly binding to the heme group of cytochrome P450-dependent enzymes of the steroidogenic pathway (Davidoff et al., 1997; Del Punta et al., 1996; Pomerantz and Pitelka, 1998). NOS is involved in testicular testosterone synthesis and causes a significant decrease in androgen production. The addition of D-Asp to incubated testicular homogenates significantly increased the testosterone concentration, whereas the addition of L-Arg decreased hormone production, suggesting an autocrine action of NO by NOS on the steroidogenic activity of Leydig cells (Lamanna et al., 2007). Furthermore, in the aging testes, treatment with either SNP or L-NAME on testicular steroidogenic factor (3-beta HSD/StAR) showed that increased NO caused decreased steroidogenesis, which is related to iNOS (Banerjee et al., 2012). Recently, intracellular mechanisms underlying the negative modulation of Leydig cell steroidogenesis by histamine (HA) have been elucidated. The anti-steroidogenic action of HA was blocked by the addition of the phospholipase C inhibitor U73122. However, the NOS inhibitor L-NAME markedly attenuated the effect of the amine on steroid synthesis. In another study, tamoxifen, as an estradiol level modulator, induced an increase in circulating steroids and testicular testosterone levels in mice after *in vivo* treatment, which may be responsible for the increased expression of testicular iNOS and consequently increased production of NO (Verma and Krishna, 2017). These findings suggest that NOS activation is the main intracellular mechanism by which HA exerts its antisteroidogenic effects.

## The role of NOS in female animal reproduction

### The role of NOS in follicle development

Follicle development is a dynamic process that is regulated by many factors, and the ovarian antral follicle count (AFC) is a marker of the ovarian stimulatory response to superovulation protocols in female animals (Alward et al., 2023). During follicular development, NO play the different roles in every stage from egg nest, different levels follicle and follicular atresia which based on microenvironment and the cross talk between granulosa cell, follicle and ovary via an intraovarian NO-generating system (Basini and Grasselli, 2015; Abdelnaby and Abo El-Maaty, 2021; Budani and Tiboni, 2021). In generally, the dual role of NO in regulating follicular development is still controversial especially follicular atresia depending mostly on its concentration and factors in reproductive system such as hormone, cytokines and others (Li et al., 2020; Cao et al., 2021; Dutta and Sengupta, 2022). Although discussed in many studies, it aslo may puzzled when defined the exactly role. Therefore, NOS may be the one of serious factor.

In the hypothalamus, nNOS potentiates adult female fertility in rodents by stimulating GnRH secretion, which, in turn, promotes luteinizing hormone release and affects follicle development by increasing nNOS activity and physiological NO during nNOS serine1412 (S1412) phosphorylation (Guerra et al., 2020). However, in the ovary, follicle development depends on the microenvironment and is based on complex factors, such as cytokines, growth factors, and locally produced substances. Many follicles are blocked and cleared because of the complex crosstalk between apoptotic cell death and cell growth signals. The activation of NO can regulate follicular development, promote the release of luteinizing hormones and gonadotropins, cause luteolysis, and induce ovulation and tissue remodeling. NOSs were expressed in many parts of the ovary in different species (Zhang et al., 2011; Li et al., 2020; Zhang et al., 2020). However, the role of the iNOS and eNOS seemed to have a double role of nitric oxide during the process of folliculogenesis and follicular which based on NO concentration and were strongly dependent on interactions with other factors acting within the ovary in a line of research (Nath and Maitra, 2019; Budani and Tiboni, 2021).

First, expression of iNOS in rat or bovine granulosa cells is accompanied by the involvement of the Fas/FasL system in inducing apoptosis through the activation of a caspase-mediated cell death (Chen et al., 2005; Zamberlam et al., 2011). However, the presence of iNOS is a requirement for immature follicles to remain quiescent, and the alteration in iNOS expression in granulosa cells of immature follicles may be a trigger, thereby rendering them atretic or developing follicles (Matsumi et al., 2000). Second, follicular development induced by pregnant mare serum gonadotropin (PMSG) in immature rats is associated with an increase in eNOS (but not iNOS) expression (Van Voorhis et al., 1995; Jablonka-Shariff and Olson, 1997), whereas subsequent stimulation with human chorionic gonadotropin (hCG) induces an increase in both isoforms (Jablonka-Shariff and Olson, 1997). In addition, the eNOS protein is localized in the cytoplasm of oocytes and theca and granulosa cells during all stages of folliculogenesis, and occasionally in the nucleus of bovine antral follicle oocytes (Pires et al., 2009; Tessaro et al., 2011).

NOS has made great progress in the female animal reproduction, since gene knockout animals have been used in the field of NO research. Furthermore, eNOS promotes primordial follicle activation, oocyte growth, and granulosa cell proliferation in neonatal ovaries via eNOS/cGMP/PKG pathway (Zhao et al., 2020). In mouse follicles cultured *in vitro*, the precursor (L-arg), intermediate (NG-OH-L-arg), and end product (L-cit) of NOS activity affected mouse follicle development. The omission of L-arginine significantly reduced follicle survival and ovulation. Partial compensation for L-arginine withdrawal was achieved using L-citrulline and NG-hydroxy-L-arginine. Specific abnormalities in follicle growth have also been reported (Mitchell et al., 2004). Supplementation of arginine, a nutritional factor, increases eNOS and sGC protein expression in theca cells and affects follicle number and cell proliferation in nutritionally compromised ewes. Therefore, the nutrition and Arg are involved in the regulation of follicular function in nonpregnant sheep (Grazul-Bilska et al., 2019). However, some studies have shown that three NOS subtypes (nNOS, iNOS, and eNOS) are expressed at the transcriptional and translational levels at different stages in buffalo follicles (PFs, AFs, and OFs). Using PMSG to induce follicular development leads to increased eNOS expression in granulosa cells. Subsequently, hCG was used to stimulate induction, and the expression of two subtypes (eNOS and iNOS) increased (Budani and Tiboni, 2021; Iova et al., 2023). Interestingly, the most significantly expressed genes encoding enzymes in the oocytes of primordial follicles differed from those expressed in oocytes at other follicular stages. The nNOS enzyme and hydroxysteroid 17-beta dehydrogenase 4, are part of the peroxisomal beta-oxidation pathway, as revealed by a genome-scale metabolic model (Peñalver Bernabe et al., 2019). However, high doses of NO induce cell death of granulosa cells and subsequently cause follicular atresia via the p38 pathway when NOS activity is elevated by some complex factors under different physiological condition (Cinelli et al., 2020; Rostamzadeh et al., 2020; Solanki et al., 2022). Nevertheless, the roles and exact mechanisms of the three NOS isoforms require further investigation.

### The role of NOS in female steroidogenesis

Among the signaling molecules that induce the function of different animal ovaries, NO is considered a regulator of steroid production (Delsouc et al., 2016). The impairment of steroid production by NOS has been demonstrated in different species and under different conditions in different species, such as humans, rats, mice, cattle, and pigs (Rosselli et al., 1998; Basini and Tamanini, 2000; Grasselli et al., 2001). In the porcine corpus luteum (CL), studies have revealed that NO produced by iNOS and eNOS not only inhibits progesterone and estradiol (E_2_) synthesis but also regulates steroidogenesis differently depending on the phase of follicular development. For example, E_2_ production in granulosa cells derived from both small (<3 mm) and medium (3–5 mm) follicles is directly inhibited by NOS via cytochrome P450 aromatase. However, in the presence of gonadotropin, NOS inhibition with methyl arginine (L-NMMA) increases the production of E_2_ and progesterone in granulosa cells, albeit to a lesser extent in less mature, small follicles than in the large mature follicles (Masuda et al., 1997; Ducsay and Myers, 2011). In contrast to earlier studies, another research group found that NO positively regulated E_2_ synthesis via cGMP during the first 24 h of culture in bovine granulosa cells, whereas it inhibited progesterone synthesis in a cGMP-independent manner. However, this surprising finding was not fully explained (Faes et al., 2009). Puzzlely, which is an isoform of NOS was involved in these negative effects and regulation of NO and E_2_ requires more studies (Hanke and Campbell, 2000; Delsouc et al., 2016). Sometimes, melatonin reverses the mRNA expression of steroidogenic enzymes and the phosphatase and tensin homolog/phosphoinositide 3-kinase/protein kinase B/mTOR/AMP-activated protein kinase signaling pathway, which reduces cyclooxygenase (Cox), particularly Cox-2, by suppressing the expression of the inducible gene of nitric oxide synthase in female rats treated with cisplatin (Al-Shahat et al., 2022). In contrast, the placenta plays a major role in steroid hormone production via placental trophoblasts. For example, progesterone synthesis in the placenta requires the involvement of a series of enzymes, including steroidogenic acute regulatory proteins (StAR), CYP11A1, and 3 beta-hydroxysteroid dehydrogenase (HSD3B). At this stage, eNOS mRNA expression and etrahydrobiopterin reduction (BH4/BH2 ratio) were increased by low-dose N-acetylcysteine. Therefore, placental progesterone levels and eNOS expression are correlated with environmental organophosphates (Rivero Osimani et al., 2016; Sanikidze et al., 2019; Ding et al., 2021; Ding et al., 2021).

### The role of NOS in ovulation

The iNOS-derived NO is required for nuclear maturation of oocytes, including germinal vesicle breakdown (GVBD) and first polar body emission in mice (Bu et al., 2004; Tao et al., 2004; Huo et al., 2005; Tao et al., 2005) and cattle (Matta et al., 2009). Some reports have demonstrated the subcellular localization of iNOS at different stages of meiotic maturation in mouse oocytes. Conversely, a reduction in iNOS expression and total nitrite levels is associated with meiotic resumption in diplotene-arrested oocytes but induces apoptosis in aged oocytes (Huo et al., 2005; Tripathi et al., 2009). In addition, the mRNA level of eNOS decreased in the follicle group 20 h after GnRH administration, followed by a rapid and significant upregulation immediately after ovulation. NO derived from iNOS also affects the *in vitro* maturation of the bovine cumulus-oocyte complex, thereby modulating the viability of cumulus cells and the oocyte. The progression of meiosis after GVBD, the migration of cortical granules, cleavage, blastocyst, and the initial phase of embryo development is an indispensable factor (Viana et al., 2007; Matta et al., 2009). In a word, NOS as paracrine factors are involved in the local mechanisms, regulating final follicle maturation, ovulation and early luteal angiogenesis (Berisha et al., 2020).

### The role of NOS in corpus luteum function

In mammals, the CL is a transient organ that secretes progesterone (P4) after ovulation, which contributes to the establishment and maintenance of pregnancy. During all development, the CL has a secretory function, and undergoes luteolysis under complex conditions, such as regression in the absence of pregnancy (Shirasuna et al., 2012; Dutta et al., 2022; Hojo et al., 2022). NO can participate in CL development and be used as a potential insertion medium to maintain angiogenesis and blood flow via three types of CL cells: steroidogenic, endothelial, and immune cells (Korzekwa et al., 2004; Weems et al., 2004; Grazul-Bilska et al., 2019; Luo et al., 2021; Dutta et al., 2022). Some studies have shown that NO inhibits P4 production, stimulates the secretion of prostaglandin (PG) F2α, reduces the number of viable luteal cells, and participates in functional luteolysis (Korzekwa et al., 2007). During this stage, eNOS and iNOS regulate NO production and include cytokines that act as pro-apoptotic and anti-apoptotic factors in bovine and rabbit CL (Motta et al., 2001; Petroff et al., 2001; Preutthipan et al., 2004; Skarzynski et al., 2005; Korzekwa et al., 2006). For instance, in the NO donor-stimulated Fas and Bax mRNA and caspase-3 expression, but not in Fas-L and bcl-2 gene expression. The ratio of bcl-2 to bax mRNA levels decreased in cells treated with the NO donor. In a recent study, NO was shown to upregulate the expression of PPARG coactivator 1 αi and its downstream factors through the cGMP pathway, thereby decreasing granulosa cell apoptosis and participating in the regulation of granulocyte steroid production through the mitochondrial-dependent pathway (Ferreira-Dias et al., 2011; Guo et al., 2019; Hojo et al., 2022).

During CL luteolysis, many reports have suggested that NO plays a crucial role in the regulation of the estrous cycle in structural luteolysis by inducing the apoptosis of luteal cells in cattle. In addition, TNF and interferon g accelerate luteolysis by increasing NO production via stimulation of iNOS expression and NOS activity in bovine luteal endothelial cells (LECs). P4 may act in maintaining CL function by suppressing iNOS expression in bovine LECs (Yoshioka et al., 2012). However, in canines, expression of eNOS and iNOS was lowest on the day of ovulation, whereas eNOS expression increased significantly towards day 20. On days 20 and 30, iNOS, endothelins exerted their vascular endothelin A receptor- and endothelin receptor B during CL rapid development-mediated effects and then activate the nitric oxide (NO) pathway (Tavares Pereira et al., 2019; Socha et al., 2022). Exogenous melatonin increased the CL diameter and colored area, accompanied by decreased NO from the serum until days 6 and 14, which may be involved in activating eNOS in heat-stressed cows (Kantar et al., 2015; Abdelnaby and Abo El-Matty, 2021). In rats, the celiac ganglion plays a physiological role in the presence of the GnRH system during luteal regression through the superior ovarian nerve at the end of pregnancy. At this stage, the release of ovarian and ganglionic NO increases. Thus, the increase in ovarian NO levels triggered by blocking ganglionic GnRH action with CTX could contribute to the increase in ovarian progesterone release and the low apoptotic luteal cell percentage observed in the experimental group, indicating that NO production by the celiac ganglion modulates the physiology of the ovary and luteal regression during late pregnancy. However, the exact mechanism underlying NOS activity remains unclear (Morales et al., 2021; Vallcaneras et al., 2022).

### NOS pathway involved in animal reproduction

The NOS pathway in animal reproduction is based on the NOS/NO system that produces NO via sGCs. NOS, a rate-limiting enzyme, uses L-arginine as a substrate and oxygen to generate NO, oxidation products, and L-citrulline (Cinelli et al., 2020; Rostamzadeh et al., 2020). NO acts *in vivo* as follows:

L-arginine +3/2 NADPH + H^+^ + 2O_2_ = citrulline + NO + 3/2 NADP and is catalyzed by NOS (Sanikidze et al., 2019; Luo et al., 2021). NO can activate sGC, a common NO sensor in mammals, through the L-arginine-NO-cGMP pathway (Hattori and Tabata, 2006; Lind et al., 2017; Iova et al., 2023). Its activation leads to the transformation of guanosine triphosphate (GTP) into cGMP, which regulates downstream cell targets, such as cGMP-dependent protein kinases, ion channels, and receptors. Although the three NOS isozymes have different structures and functions, they share similar pathways in NO synthesis (Luo et al., 2021; Dutta and SenGupta, 2022).

**FIGURE 4:**
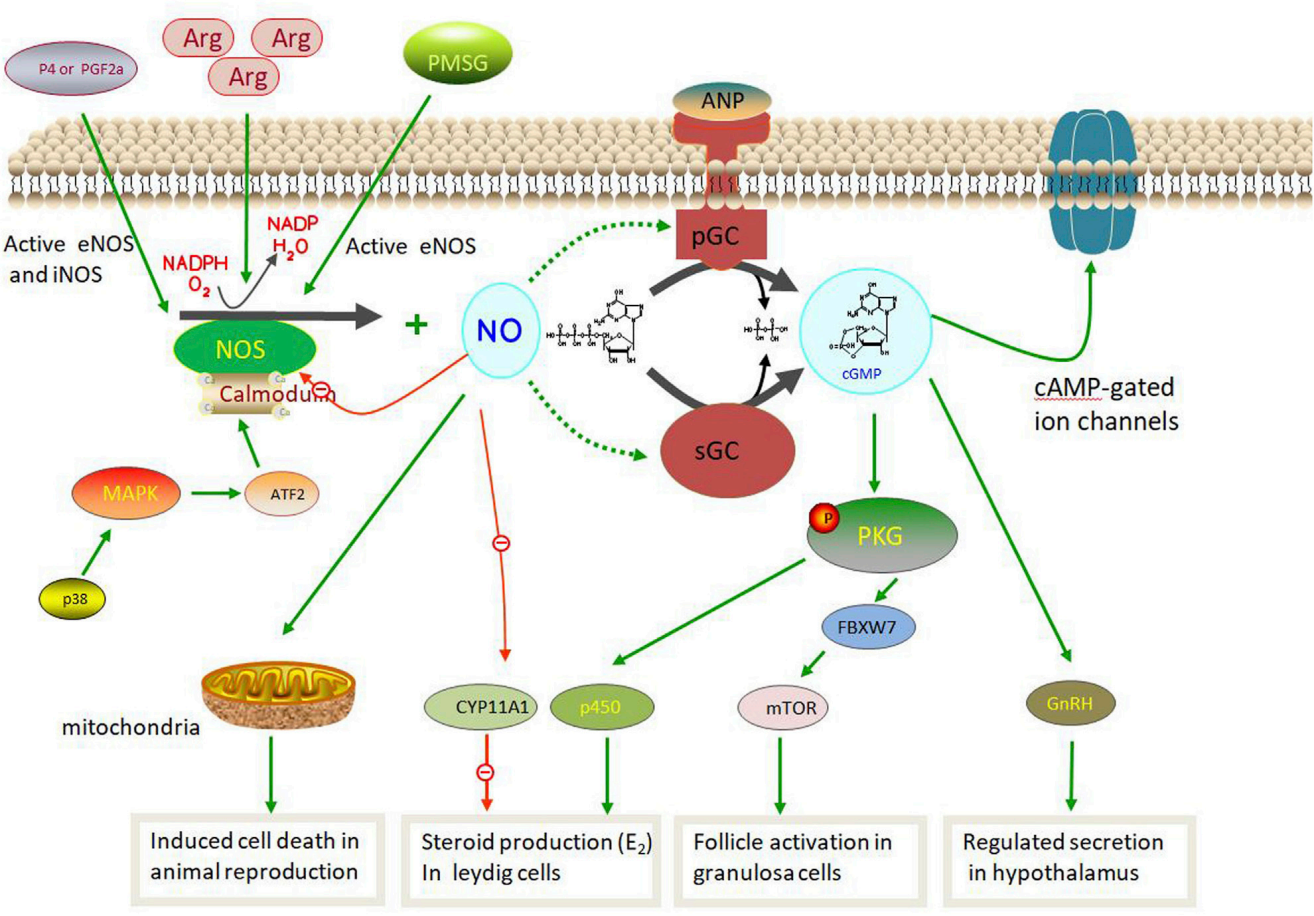
Nitric oxide synthase (NOS) pathway involved in animal reproduction.

During this process, NO induces rupture of the His–Fe (II) bond within the heme of sGC, a heterodimer composed of one α (α1 or α2) and a βsubunit (β1). This induces a conformational change in the His ligand (pentacoordinated NO complex), which is conveyed to the catalytic center in a partially obscure manner, resulting in increased activity for the conversion of GTP to cGMP (Francis et al., 2010; Hall and Garthwaite, 2009; Martínez-Ruiz et al., 2011; Russwurm and Koesling, 2004). Several pathways play important roles in animal reproduction (Figure 4). In the hypothalamic and pituitary gonadotropes, NOS plays an important role in controlling reproduction owing to its ability to control GnRH secretion (Mondillo et al., 2009). Moreover, iNOS protein expression and activity were increasing via p38 MAPK/ATF2/iNOS signaling pathway involved in testicular injury induced by HgCl_2_ as in inflammation condition which leading spermatogenetic activity, seminiferous tubular diameter, and Leydig cell mass (Sangodele et al., 2021; Chen et al., 2022). Similar in follicular atresia, NO produced by iNOS aslo can play the negatively affected cell death via the p38 pathway during follicle development. Therefore, iNOS is involved in testicular damage and cell death via decreased tissue antioxidative defense capacity and the regulation of testicular hemodynamics **(** El-Shalofy et al., 2023). In male animals, NO produced by NOS can activate cGMP-dependent PRKG1 and phosphorylate astrocytes to activate soluble cGMP, thus promoting steroid production in Leydig cells. However, NO can inhibit CYP11A1 at high doses and inhibit steroid production (Wei et al., 2002; Mondillo et al., 2009). However, in females, exogenous ovarian nerves can directly control NO and E_2_ levels during the follicular phase (Rosselli et al., 1998; Basini and Tamanini, 2000; Grasselli et al., 2001). Furthermore, eNOS promoted primordial follicle activation, oocyte growth and granulosa cell proliferation in neonatal ovaries via the eNOS/cGMP/PKG pathway and then worked on FBXW7 and mTOR protein induced primordial follicle activation (Mitchell et al., 2004; Tessaro et al., 2011; Zhao et al., 2020). Notably, PMSG can activate eNOS expression, which in turn induces follicular development in granulosa cells (Budani and Tiboni, 2021; Iova et al., 2023). However, the new NOS pathway in animal reproduction requires further exploration.

## Conclusion

Over the past 30 years, NOS, the producer of NO in the body, acted as three isoforms and play essential role via abundant location in animal reproduction which also hot spot today. NOS regulated NO’s dual and dynamic role in male and female reproductive functions via many factors in reproductive system such as hormone, cytokines and others. Notable, NOS are associated with the injuries, pathology and abnormal condition of physiological processes during animal reproduction. In turn, the more discovery about the structure of NOS and relations pathway will help to make clear the exactly mechanisms. As can be seen in this review, the various biological functions of reproduction associated with NOS can affect organs or systems. However, many processes in the NOS have still not been fully explained yet. Focusing on these topics can help provide the new insight into and the treatment evidence on targeting NOS of reproductive regulation and diseases. Therefore, it is foreseeable that the continuous development of the study of NOS and its mechanism will have broad prospects in the future.

## Abbreviations

AFs, antral follicles; BH4, tetrahydrobiopterin; CaM, calmodulin; cryo-EM, cryogenic electron microscopy; dNOS, *Drosophila* nitric oxide synthases; eNOS, endothelial nitric oxide synthases; FAD, flavin-adenine dinucleotide; FMN, flavin-adenine mononucleotide; HSP90, heat shock protein 90; iNOS, inducible nitric oxide synthases; L-NAME, L-arginine methyl ester; LPS, lipopolysaccharide; mtNOS, mitochondrial nitric oxide synthases; NADPH, nicotinamide adenine dinucleotide phosphate; nNos, neuronal nitric oxide synthases; NO, nitric oxide; PnNOS, penile nitric oxide synthases; TnNOS, testis-specific nitric oxide synthases.
